# Quantitative liquid chromatography-tandem mass spectrometric analysis of 11dH-TXB2 and creatinine in urine: Determination of 11dH-TXB2 and creatinine levels using LC-MS/MS

**DOI:** 10.3724/abbs.2025055

**Published:** 2025-07-18

**Authors:** Chunyan Li, Wuzheng Liu, Yana Xiao, Tenglong Dai, Yu Su, Yubin Wang, Ao Zhang, Ruichen Liu, Xianglong Zhao, Zhao Zhang, Shangqi Yin, Jun Wu

**Affiliations:** 1 Department of Clinical Laboratory Beijing Jishuitan Hospital Capital Medical University Beijing 100035 China; 2 Department of Laboratory Medicine Peking University Fourth School of Clinical Medicine Beijing Jishuitan Hospital Beijing 100035 China; 3 Genetic Metabolism Medical Center Dalian Women and Children’s Medical Center (Group) Dalian 116021 China; 4 Shanghai AB Sciex Analytical Instrument Trading Co. Ltd. Beijing 100015 China; 5 Peking University Fourth School of Clinical Medicine Beijing 100191 China

Platelets circulate in an inactive form in the body until they contact with defective areas of endothelial cells or encounter a clotting cascade [1]. Activated platelets release and express bioactive substances and acquire the ability to bind plasma fibrinogen. Abnormal activation of platelets is involved in atherosclerosis and thrombosis [ 2, 3]. When platelets are stimulated and activated, phospholipase A2 is activated at the same time, which then cleaves membrane phospholipids and frees arachidonic acid (AA) [4]. The latter catalyzes thromboxane A2 (TXA2) via thromboxane synthetase [5], which is induced by the cyclooxygenase COX-1 to produce prostaglandins G2 and H2 [6]. TXA2 is highly unstable, with a half-life of only 30 s, and it is rapidly hydrolyzed to relatively stable thromboxane B2 (TXB2), which is then converted in the liver to 11-dehydrothromboxane B2 (11dH-TXB2), which has a longer half-life and is excreted in the urine [7]

Dehydrothromboxane B2 is the final stable metabolite of thromboxane A2, which is derived only from arachidonic acid metabolism and can represent thromboxane A2 level in the body [8]. Specifically, by inhibiting the action of COX-1, the most important enzyme in the process of arachidonic acid metabolism, aspirin inhibits the production of thromboxane A2, that is, the concentration of TXA2 affects the effect of aspirin on platelet aggregation. However, the half-life of TXA2 (including the metabolic intermediate TXB2) is too short to be accurately measured, so the detection of its metabolic end product 11dH-TXB2 can very accurately reflect the sensitivity of the body to aspirin [7].

The concentration of 11dH-TXB2 in the serum correlates well with the concentration of 11dH-TXB2 in the urine, so the determination of 11dH-TXB2 in the urine can more effectively reflect the production of TXA2 *in vivo* [8]. The 11dH-TXB2 concentration needs to be corrected with the urinary creatinine concentration to rule out the effects of the urine concentration and renal function, so random urine samples can be used for testing [6].

Aspirin can acetylate serine at the key site of cyclooxygenase and thus irreversibly inhibits the activity of COX-1, reduces the synthesis of TXA2, and blocks the production of TXA2 and its induced platelet aggregation. Low-dose aspirin (30–75 mg/day) can effectively inhibit 95% of COX-1 activity [7]. Since the production of TXA2 in serum is largely dependent on platelet COX-1 (a therapeutic target of aspirin), 11dH-TXB2 can be used as a monitor for aspirin-induced platelet inhibition [3].

Creatinine is a metabolic byproduct of muscle metabolism that is primarily excreted via glomerular filtration, and its level is indicative of renal function [9]. The 24-h creatinine clearance can also be used to determine the integrity of the sample or to correct the urine sample concentration with the creatinine ratio [10]. Currently, creatinine detection methods include the Jaffe method, enzymolysis spectrophotometry, HPLC, capillary electrophoresis, capillary zone electrophoresis, gas chromatography tandem mass spectrometry (GC-MS) and liquid chromatography tandem mass spectrometry (LC-MS/MS) [11].

At present, there is no method for the simultaneous detection of 11dH-TXB2 and creatinine. When both analytes are needed, separate tests must be performed, increasing the workload and sample volume requirements. Thus, developing a method that enables the concurrent quantification of 11dH-TXB2 and creatinine in a single assay remains a critical challenge.

The aim of this study was to provide a method for the simultaneous detection of 11dH-TXB2 and creatinine and to alleviate the problem that 11dH-TXB2 and creatinine cannot be simultaneously detected. By developing a standardized quantitative approach for measuring 11dHTXB2 and creatinine in human urine, this study aims to provide reliable concentration data, thereby facilitating further clinical research and methodology optimization.

Multiple samples with low urine 11dH-TXB2 and creatinine background values were mixed and used as a blank matrix for methodological validation. The corresponding concentrations of 11dH-TXB2 and creatinine standard solutions were added to the blank matrix of urine, urine quality control samples with low, medium and high concentrations were prepared, and procedures were followed for “sample pretreatment”. An 11dH-TXB2 stock solution was prepared by dissolving 10 mg powder to 10 mL pure water:methanol:acetonitrile (8:1:1) to obtain a concentration of 1 mg/mL. The creatinine stock solution was prepared by dissolving 10 mg powder to 10 mL pure water to obtain a concentration of 1 mg/mL. The linear working solutions were prepared by diluting the stock solutions of 11dH-TXB2 and creatinine with PBS. The concentrations of 11dH-TXB2 were: 50 ng/mL, 25 ng/mL, 10 ng/mL, 2 ng/mL, 0.5 ng/mL, 0.2 ng/mL, and 0.1 ng/mL; the concentrations of creatinine were: 5000 ng/mL, 2500 ng/mL, 1000 ng/mL, 200 ng/mL, 50 ng/mL, 20 ng/mL, and 10 ng/mL. The mixed internal standards of 11dH-TXB2 and creatinine were prepared by diluting the stock solutions with PBS to a final concentrations of 50 ng/mL and 1 μg/mL respectively. In the same batch, 6 samples were prepared and analyzed for each concentration, and the variation in the batch was investigated. In accordance with the above method, 3 different batches of urine samples with 3 concentrations (low, middle and high) were prepared, and the standard curve prepared on the same day was analyzed and determined to investigate the inter-batch variation. Ethical approval was provided by the Ethics Committee of Beijing Jishuitan Hospital (Beijing, China).

The accuracy of the method was investigated according to the degree of closeness of the measured concentration of the sample and the theoretical real concentration. The results are shown in Table 1. The test results indicate that the accuracy of all the quality control samples is within the error range and that all the samples are included in the statistics. The accuracy of all 3 quality control samples with different concentrations ranged from 85.83% to 113.21%, and the Relative Standard Deviation (RSD%) values of the quality control samples were all larger than 9.71%. These results show that this method is stable and reliable and meets the requirements of human urine sample detection.

**Table:** **Table 1** 11dH-TXB2 and creatinine intra-lot and inter-lot precision and accuracy data2

Analyte	Batch	L		M		H
RSD%	Accuracy		RSD%	Accuracy		RSD%	Accuracy
11dH-TXB2	The first batch	8.80	97.19		5.16	93.67		3.50	89.61
The Second batch	9.71	102.07		7.55	89.16		2.48	85.83
The third batch	3.19	90.08		3.51	102.78		7.69	106.91
Creatinine	The first batch	3.59	98.39		4.05	90.07		2.40	92.52
The second batch	5.89	99.60		5.34	88.33		6.53	100.08
The third batch	8.89	113.21		9.25	105.12		5.13	98.82

The degree of chromatographic separation of 11dH-TXB2 and creatinine from the interference peak in the matrix was tested. The results revealed that the separation degree was greater than 1.5, and the interference around the peak time of 11dH-TXB2 and creatinine did not affect the quantitative analysis. Chromatograms of 11 dH-TXB2 and creatinine in the substitute substrates, standard solution, human urine, and blank solution were compared, and the results are shown in Figure 1.

**Figure 1:**
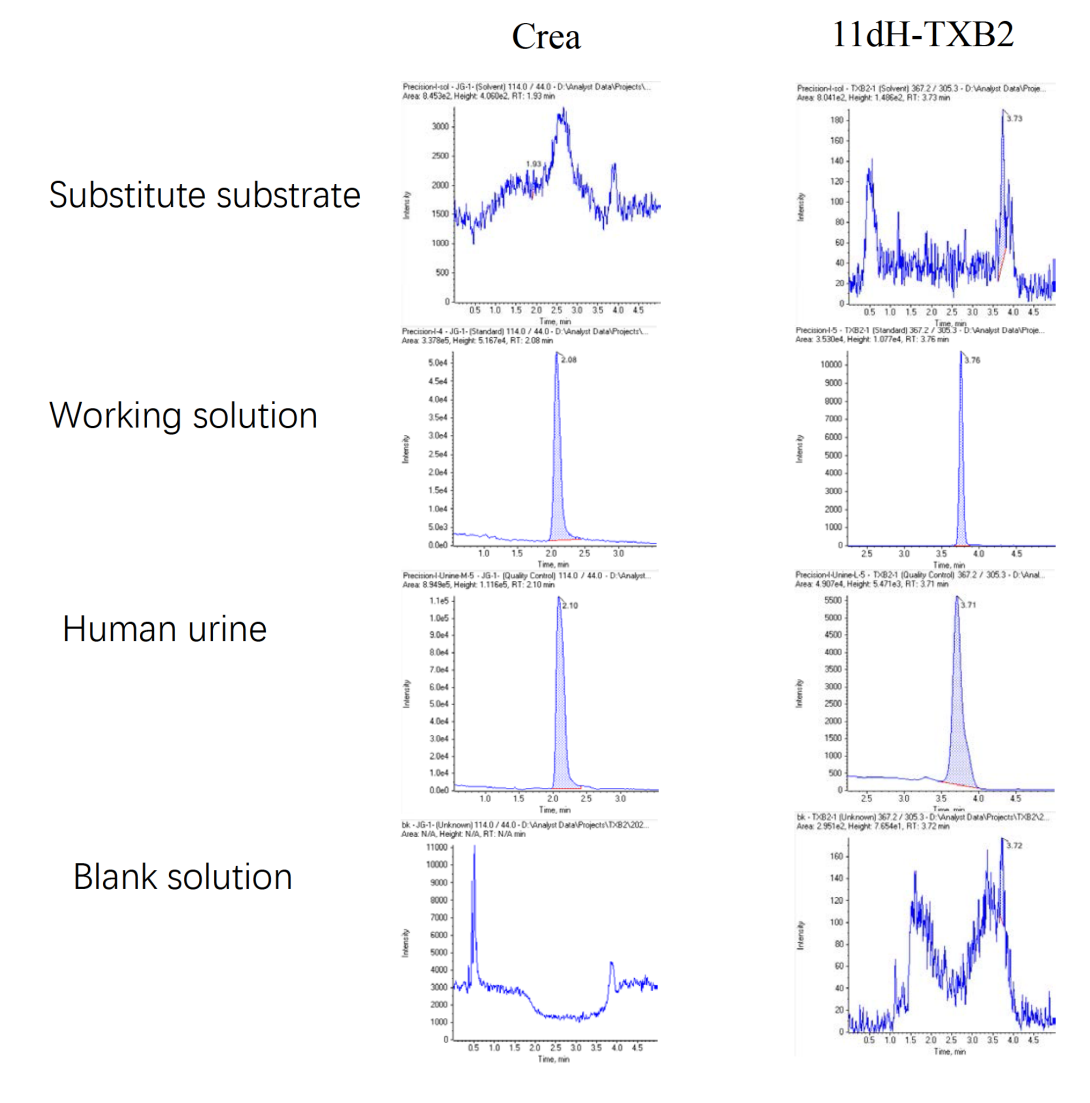
Figure 1 Chromatograms of 11dH-TXB2 and creatinine in various sample types This figure compares chromatographic profiles of 11dH-TXB2 and creatinine across four sample types: substitute substrate, standard working solution, human urine, and blank solution. Results demonstrate effective chromatographic separation (separation degree > 1.5), with minimal interference at the peak times of 11dH-TXB2 and creatinine, confirming reliability for quantitative analysis. 11dH-TXB2: 11-dehydrothromboxane B2; Crea: creatinine.

The replacement substrate is PBS with 7 relative concentrations of 11dH-TXB2 and creatinine. Five samples were prepared and analyzed in the same run. The allowable deviation of linearity is ± 15% of the true value. With the concentrations of 11dH-TXB2 and creatinine as the horizontal coordinates and the ratio of the peak area to the internal standard peak area as the vertical coordinate, regression equations were obtained, as shown in Table 2.

**Table:** **Table 2** 11dH-TXB2 and creatinine linear range data

Analyte	Linearity
Linear equation	Correlation coefficient r ^2^
11dH-TXB2	y = 0.09971x + 0.00348	0.99656
Creatinine	y = 0.01877x + 0.00151	0.99950

According to Clinical and Laboratory Standards Institute (CLSI) guidelines, at 11dH-TXB2 concentrations of 0.25 ng/mL, 5 ng/mL, and 12.5 ng/mL, the recovery rate and matrix effect were calculated at creatinine concentrations of 25 ng/mL, 500 ng/mL and 1250 ng/mL. Three kinds of mixed quality control working solutions with different concentrations were prepared and added to the urine sample to prepare the quality control sample in triplicate. Group A contained a pure water solution, and groups B and C contained low-value urine.

The working solution was blended into groups A and C. Sample preparation was performed for groups B and C. After the supernatant was extracted, Group B was reformulated with the working solution.

The area ratio of the analyte in group C was calculated to that in group B at the corresponding concentration, and the relative recovery rate of the analyte was obtained. The area ratio of group B analytes was calculated to determine the area ratio of group A analytes at the corresponding concentration to obtain the matrix effect of the analytes as shown in Supplementary Table S1.

Residues were evaluated by injecting samples and blanks containing 11dH-TXB2 and creatinine at the highest linear concentration points. During validation, samples were injected following the CLSI-recommended concentration order (medium, high, low, medium, medium, low, low, high, high, and medium). The residues were required to be < 20% of the analyte’s LLOQ and < 5% of the isotope internal standard. The results confirmed the absence of interference.

Urine quality control samples were prepared by adding low-background urine to quality control solutions at three concentrations (L, M, and H). These samples were then used to investigate stability under two conditions: (1) storage at room temperature for 6 h and (2) storage in preprocessed form in an autosampler at cold storage for 6 h. The results are summarized in Supplementary Table S2.

Urine quality control samples were prepared by separately spiking 11dH-TXB2 and creatinine standard solutions at the lowest quantitation concentration into a blank urine matrix. The samples were processed following the “sample pretreatment” protocol. Six replicates were prepared and measured to evaluate the variability at the lower limit of quantification (LLOQ). The results are detailed in Supplementary Table S3.

Selection and optimization of chromatographic conditions: The effects of different salt solutions (ammonium formate and ammonium fluoride) and the ratio of ammonium formate buffer (0.1 mM, 0.2 mM and 1 mM) in the mobile phase on chromatographic separation were investigated. The results revealed that when the ratio of ammonium formate buffer solution in mobile phase A was 0.2 mM, the chromatographic separation effect was good, the baseline was stable, the peak shape was symmetrical, and the separation degree was good. Moreover, the addition of ammonium formate can significantly increase the stability of the chromatographic peak retention time, so the mobile phase was determined as follows: phase A (aqueous solution containing 0.2 mM ammonium formate)-phase B (methanol solution containing 0.2 mM ammonium formate). After the elution ratio of the mobile phase was adjusted at different time points, the retention time of each chromatographic peak was moderate, and the baseline was stable, which was not easy to drift. Moreover, the separation degree of the chromatogram is improved, and the tailing phenomenon of the chromatogram is effectively avoided, which is conducive to the detection and analysis of 11dH-TXB2 and creatinine. As shown in Figure 2, the above method was applied to detect the concentrations of 11dH-TXB2 and creatinine in the urine of 8 patients. The concentrations of 11dH-TXB2 and creatinine in the urine were measured, as shown in Supplementary Table S4.

**Figure 2:**
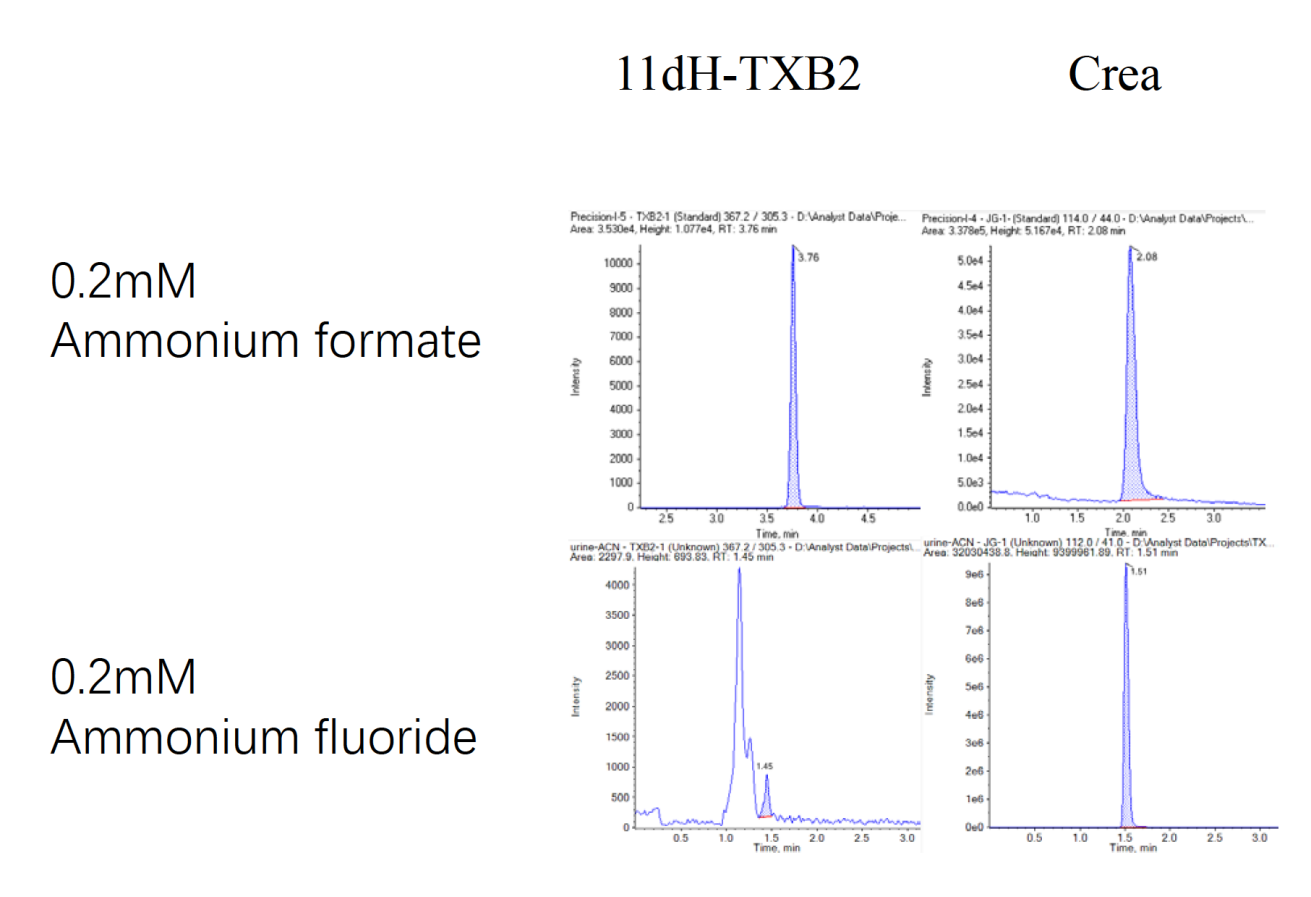
Figure 2 Chromatographic separation of 11dH-TXB2 and creatinine with different buffer solutions This figure compares the chromatographic separation of 11dH-TXB2 and creatinine using mobile phases containing 0.2 mM ammonium formate (top) and 0.2 mM ammonium fluoride (bottom). The chromatograms demonstrate that the use of ammonium formate yields stable baselines, symmetrical peaks, and effective separation for both compounds. In contrast, ammonium fluoride results in less stable baselines and peak shapes, highlighting the superior separation performance of ammonium formate for the detection and analysis of 11dH-TXB2 and creatinine. 11dH-TXB2: 11-dehydrothromboxane B2; Crea: creatinine.

The 11dH-TXB2 and creatinine concentrations in human urine samples were determined via established and confirmed analytical methods, and the results of the samples to be measured were accurate and reliable.

By using the established quantitative methodology for 11dH-TXB2 and creatinine in human urine, the concentration data of 11dH-TXB2 and creatinine in urine samples were obtained to provide data support and methodology reference for subsequent clinical studies [11].

In summary, the present method can simultaneously detect the contents of 11dH-TXB2 and creatinine in samples. After the samples are pretreated, the two analytes of 11dH-TXB2 and creatinine can be detected by liquid chromatography and mass spectrometry, which has the advantages of convenience and speed. In addition, the pre-processing steps of the method described in this paper are simple and require only the organic phase to extract the analyte from the sample, without complex operations such as solid-phase extraction, further simplifying the detection method. Our method has the characteristics of high sensitivity, good repeatability, high accuracy and good specificity. In the optimal scheme, the linear range of 11dH-TXB2 was 0.1-50 ng/mL, the linear range of creatinine was 10–5000 ng/mL, and the correlation coefficients (r ^2^) in the above linear ranges were all ≥ 0.990. The accuracies of the low-value, median-value and high-value quality control products were between 85.83% and 113.21%, the RSDS% values of the quality control samples were less than 9.71%, and the recoveries of the standard products were 85% to 110%. This method meets regulatory requirements for linearity, repeatability, inter-batch variability, and accuracy in the simultaneous quantification of 11dH-TXB2 and creatinine. This approach holds significant potential for investigating the pathological mechanisms of related diseases and facilitating the development of novel therapeutics.

## Supporting information

24964supplementary_Tables

## Supplementary Data

Supplementary Data is available online at *Acta Biochimica et Biophysica Sinica*.
