# Supplementary material for: Quantitative liquid chromatography-tandem mass spectrometric analysis of 11dH-TXB2 and creatinine in urine: Determination of 11dH-TXB2 and creatinine levels using LC-MS/MS
Source: Acta Biochim Biophys Sin (Shanghai). 2025 Jul 18;57(12):2124–8. doi: 10.3724/abbs.2025055 (PMC12747966; doi:10.3724/abbs.2025055)
Supplement: 24964supplementary_Tables [file 24964supplementary_Tables.docx]

**Supplementary Table S1. 11dH-TXB2 and creatinine relative recovery and matrix effect data**

| Analyte | Relative recovery | | | Matrix effect | | |
| --- | --- | --- | --- | --- | --- | --- |
|  | L | M | H | L | M | H |
| 11dH-TXB2 | 102.83 | 92.17 | 88.74 | 109.80 | 99.68 | 96.85 |
| Creatinine | 97.91 | 85.13 | 96.64 | 97.23 | 98.13 | 98.47 |

**Supplementary Table S2**. **11dH-TXB2 and creatinine stability data**

| Analyte | Room temperature stability (CV) | | | Cold storage stability (CV) | | |
| --- | --- | --- | --- | --- | --- | --- |
|  | L | M | H | L | M | H |
| 11dH-TXB2 | 99.96 (6.37) | 90.64 (1.99) | 91.34 (2.87) | 101.17 (6.44) | 92.38 (5.43) | 92.55 (2.73) |
| Creatinine | 100.24 (1.99) | 92.83 (5.13) | 93.46 (2.41) | 98.65 (4.6) | 98.36 (2.53) | 86.26 (3.27) |

**Supplementary Table S3**. **11dH-TXB2 and creatinine minimum quantification data**

| Analyte | Minimum quantitative limit (CV) |
| --- | --- |
| 11dH-TXB2 | 106.09 (3.11) |
| Creatinine | 91.38 (3.80) |

**Supplementary Table S4**. **Urine concentrations of 11dH-TXB2 and creatinine in 8 cases**

| Sample name | Determination of 11dH-TXB2 by mass spectrometry (ng/mL) | Determination of creatinine by mass spectrometry (μg/mL) |
| --- | --- | --- |
| Unine-1 | 6.45 | 390.27 |
| Unine-2 | 2.53 | 396.31 |
| Unine-3 | 2.74 | 471.78 |
| Unine-4 | 3.44 | 641.25 |
| Unine-5 | 4.55 | 953.66 |
| Unine-6 | 3.41 | 269.02 |
| Unine-7 | 28.19 | 517.05 |
| Unine-8 | 6.77 | 451.48 |
